# Paradoxical pericarditis in an immunocompetent patient with mediastinal tuberculous lymphadenitis and endobronchial TB: A case report

**DOI:** 10.1016/j.idcr.2025.e02363

**Published:** 2025-09-12

**Authors:** Tomohiro Oba, Hidekazu Matsushima, Masako Amano, Keiichi Akasaka, Tomotaka Nishizawa

**Affiliations:** Department of Respiratory Medicine, Saitama Red Cross Hospital, Saitama, Japan

**Keywords:** Paradoxical reaction, Pericarditis, Tuberculosis, Lymphadenitis, Endobronchial tuberculosis

## Abstract

**Background:**

Paradoxical reaction (PR) refers to a worsening of existing or new TB-related lesions after initiating effective anti-tuberculosis therapy. While commonly observed in lymph node or CNS TB, PR involving the pericardium is exceedingly rare in immunocompetent patients.

**Case presentation:**

A 22-year-old Vietnamese man with mediastinal tuberculous lymphadenitis and endobronchial TB was started on standard anti-TB therapy. After initial improvement, he developed pericardial effusion three months into treatment. Cultures and PCR were negative, but elevated ADA levels were detected. Based on clinical course and exclusion of treatment failure or coinfection, paradoxical pericarditis was diagnosed. Corticosteroids and levofloxacin were added empirically, with resolution of symptoms.

**Conclusion:**

This case highlights a rare extrapulmonary manifestation of PR, reinforcing the need to distinguish it from treatment failure or TB progression in immunocompetent hosts.

## Introduction

Paradoxical reaction (PR) is defined as the clinical or radiologic deterioration of existing TB lesions or the development of new lesions in patients who initially respond to appropriate anti-TB therapy. Although PR is well recognized in HIV-positive patients, it is also well documented in HIV-negative individuals [1], [2]. PR most commonly involves lymph nodes and the central nervous system [3], while pericardial involvement is rare, particularly in immunocompetent patients. Only a limited number of such cases have been reported [4], [5]. We present a case of PR-associated pericarditis in a young immunocompetent adult with mediastinal tuberculous lymphadenitis and endobronchial TB, highlighting diagnostic challenges and therapeutic considerations.

## Case presentation

**Fig. 1:**
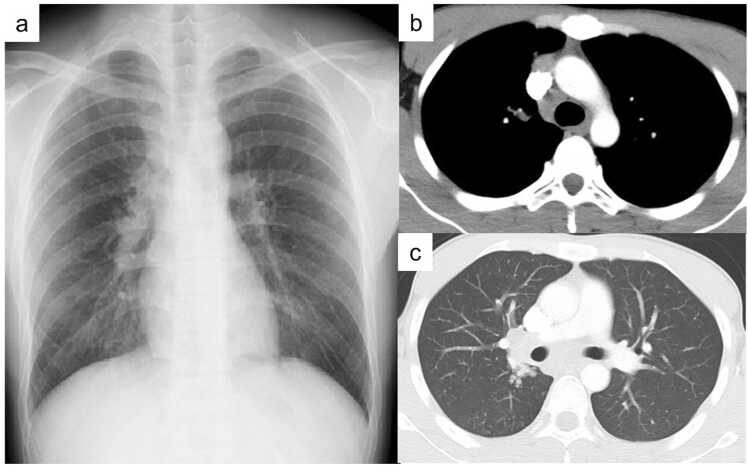
Chest X-ray showing mediastinal widening at presentation (a). Chest CT before treatment demonstrating enlarged mediastinal lymph nodes (b) and pericardial effusion (c).

**Fig. 2:**
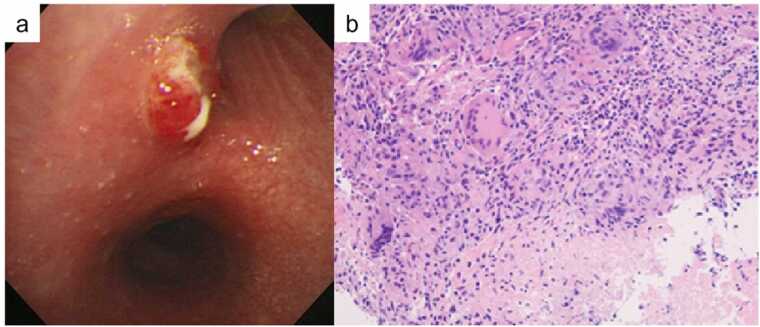
Bronchoscopic view of a nodule at the inlet of the right upper lobe (a). Histopathological image showing epithelioid cell granuloma with central necrosis (H&E stain, ×100) (b).

A 22-year-old Vietnamese man with no past medical history was referred as a TB contact. He denied fever, cough, chest pain, or weight loss. Physical examination was normal. Baseline laboratory tests, including complete blood count, liver and renal function, C-reactive protein, and HIV antibody, were within normal limits. Screening chest CT revealed enlarged mediastinal lymph nodes (Fig. 1). EBUS-TBNA from a subcarinal node showed caseating granulomas, and biopsy of an endobronchial lesion revealed necrotizing granulomatous inflammation (Fig. 2). Both tissue culture and PCR for *M. tuberculosis* were positive, with no resistance mutations detected.

Standard four-drug therapy—isoniazid, rifampicin, ethambutol, and pyrazinamide—was initiated according to ATS/CDC/IDSA guidelines [6]. After 12 weeks of treatment, while still on this regimen, he developed acute fever (38.5°C) and progressive dyspnea. On examination, he had tachycardia (110 bpm), reduced heart sounds, and mild jugular venous distension, without pericardial friction rub. Laboratory studies showed elevated CRP (8.2 mg/dL) and ESR (85 mm/h), normal white blood cell count, and negative HIV serology.

**Fig. 3:**
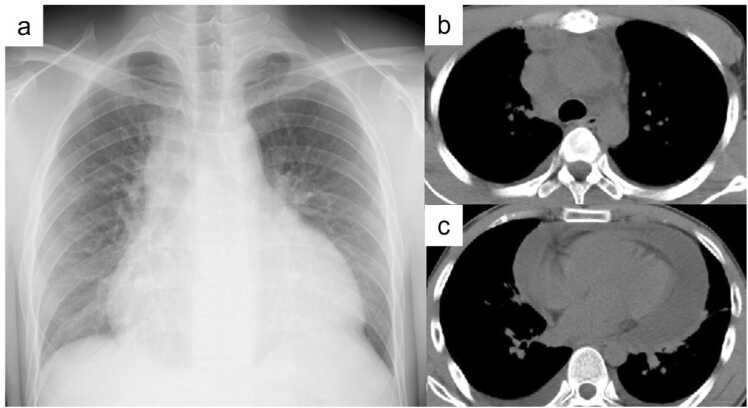
Chest X-ray after initiation of anti-tuberculosis therapy, showing improvement of mediastinal widening (a). Chest CT after three months of treatment revealing reduction of mediastinal lymphadenopathy (b) and pericardial effusion (c).

Chest CT demonstrated re-enlargement of mediastinal lymph nodes, new bronchopneumonia in the right middle lobe, and moderate pericardial effusion (Fig. 3). Pericardiocentesis revealed lymphocyte-predominant exudate with ADA of 85.8 IU/L; PCR and cultures for *M. tuberculosis* and common bacterial pathogens were negative.

Given the temporal relationship to therapy, exclusion of treatment failure or new infection, and adherence to WHO diagnostic criteria for PR [7], paradoxical pericarditis was diagnosed. Due to the patient’s hemodynamic symptoms and radiologic findings, empirical levofloxacin was added for possible bacterial co-infection, despite awareness of its second-line role in TB therapy and potential resistance risk. Oral corticosteroids (prednisolone 0.5 mg/kg/day) were started concurrently.

**Fig. 4:**
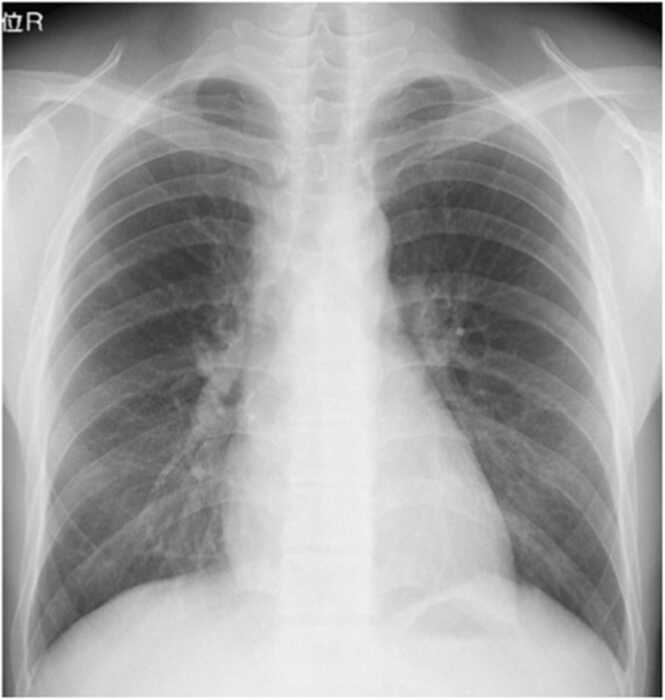
Chest X-ray after 1 month of steroid therapy showing improvement of pericarditis and mediastinal lymphadenitis.

The patient’s symptoms improved within days, and repeat chest radiography after one month showed marked reduction in pericardial effusion and mediastinal lymphadenopathy (Fig. 4). Unfortunately, the patient did not return for further follow-up, limiting assessment of long-term outcomes.

## Discussion

Paradoxical reaction is differentiated from treatment failure by negative cultures, absence of drug resistance, and the presence of clinical or radiological worsening during otherwise effective anti-tuberculosis (TB) therapy [1], [3]. In this patient, the absence of active TB in the pericardium and the rapid improvement after corticosteroid therapy supported the diagnosis of paradoxical pericarditis rather than tuberculous pericarditis.

Corticosteroid therapy in tuberculous pericarditis remains controversial. Historically, adjunctive corticosteroids have been used to suppress pericardial inflammation, aiming for rapid symptom relief in the acute phase and prevention of late complications such as constrictive pericarditis and death [5], [6]. Early studies, such as the randomized trial by Strang et al., suggested reductions in morbidity and mortality [8]. However, subsequent evidence has been mixed. The large, randomized Investigation of the Management of Pericarditis (IMPI) trial demonstrated that prednisolone reduced the incidence of constrictive pericarditis but did not significantly improve overall survival in HIV-negative patients, and showed an increased incidence of HIV-related malignancies in HIV-positive patients [9]. The 2017 Cochrane systematic review concluded that corticosteroids probably reduce pericarditis-specific mortality but have uncertain effects on all-cause mortality and other outcomes, particularly in people living with HIV [10].

Consequently, the 2015 European Society of Cardiology (ESC) guidelines on pericardial diseases [11] state that adjunctive corticosteroids may be reasonable in HIV-negative patients but should be avoided in HIV-positive patients because of malignancy risk. The 2022 WHO consolidated guidelines on TB treatment [12] allow corticosteroid use for tuberculous pericarditis (“recommended” or “may be used” depending on section wording) but do not advocate routine universal use. Both emphasize individualized consideration based on disease severity and patient factors.

Important unresolved issues include the optimal timing of initiation (e.g., concomitant with anti-TB therapy versus upon clinical deterioration), the appropriate dosing regimen, and the total duration of therapy. In clinical trials, prednisolone was commonly initiated at 60 mg/day (approximately 1 mg/kg/day) and tapered over 6–8 weeks [9], [10]. In the present case, prednisolone was initiated at 0.5 mg/kg/day shortly after the diagnosis of pericarditis due to paradoxical reaction, in conjunction with ongoing standard anti-TB therapy and levofloxacin, leading to prompt improvement. This favorable outcome supports the view that corticosteroids can be beneficial in selected patients, particularly those with severe inflammatory manifestations despite appropriate anti-TB treatment.

## Limitations

This case report has several limitations. Histopathological confirmation of pericarditis was not obtained, and although microbiologic studies were negative, paucibacillary tuberculous pericarditis could not be entirely ruled out. Levofloxacin and corticosteroids were started concurrently, limiting assessment of their individual effects. Empirical levofloxacin was added due to clinical severity and concern for bacterial coinfection. Follow-up data were unavailable due to patient discontinuation of outpatient visits, preventing assessment of late complications such as constrictive pericarditis. The diagnosis was also based on criteria described in a widely used tuberculosis reference text [10].

## Conclusion

PR should be considered in patients who clinically worsen despite adequate therapy, especially when other causes are ruled out. Pericardial involvement, although rare, may be underdiagnosed and requires prompt recognition and immunomodulatory treatment in select cases.

## Author statement

All authors have read and approved the final manuscript.

No specific funding was received for this work.

Written informed consent was obtained from the patient for publication of this case report and any accompanying images.

The authors confirm that the work described has not been published previously, is not under consideration for publication elsewhere, and complies with the ethical standards of the responsible committee on human experimentation.

## CRediT authorship contribution statement

**Tomotaka Nishizawa:** Data curation. **Masako Amano:** Data curation. **Keiichi Akasaka:** Data curation. **Tomohiro Oba:** Writing – original draft, Conceptualization. **Hidekazu Matsushima:** Data curation.

## Declaration of Competing Interest

The authors declare that they have no known competing financial interests or personal relationships that could have appeared to influence the work reported in this paper.
